# Acute Abdominal Pain as a Result of an Isolated Left Ovarian Vein Thrombosis

**DOI:** 10.1155/2023/9528088

**Published:** 2023-04-22

**Authors:** Abhay Setia, Farzin Adili, Karl Ludwig, Joerg Herold

**Affiliations:** ^1^Department of Vascular Medicine, Division of Vascular and Endovascular Surgery, Klinikum-Darmstadt, Darmstadt, Germany; ^2^Department of Radiology, Neuroradiology and Nuclear Medicine, Klinikum-Darmstadt, Darmstadt, Germany; ^3^Department of Vascular Medicine, Division of Angiology, Klinikum-Darmstadt, Darmstadt, Germany

## Abstract

Ovarian vein thrombosis (OVT) is a rare thromboembolic condition. It involves the right ovarian vein in 70–80% of cases. The risk factors for the development of OVT are pregnancy or puerperium, hormone therapy with estrogen, recent surgery or hospitalization, malignancy, pelvic inflammatory diseases, thrombophilia and idiopathic OVT. We present a rare case of left OVT in a young, non-pregnant woman in her 30 s. A high degree of suspicion is necessitated in patients with the triad of young-middle-aged female, pain abdomen in lower quadrant and hematuria to diagnose OVT. Contrast enhanced computer tomography (CT-venography) is the diagnostic modality of choice. The patient was initially treated with low molecular weight heparin and then switched to direct oral anticoagulants. At 6-monthsfollow-up the patient was free from any symptoms.

## 1. Background

With 70–80% of the ovarian vein thrombosis (OVT) involving the right ovarian vein (OV), thrombosis of the left OV is a yet rare thromboembolic condition with unknown incidence in the general population [1]. The postpartum (usually 2–6 days postpartum) or septic puerperal ovarian vein thrombosis has an incidence of 1 : 600–6000, in combination of caesarean births. In the case of a septic course, it can be life-threatening. Out of the multiple risk factors, thrombophilia is reported in up-to 24% of patients in various case series [2–5]. We portray a case of isolated left ovarian vein thrombosis presenting with abdominal pain in left lower quadrant (LQ).

## 2. Case Report

**Figure 1:**
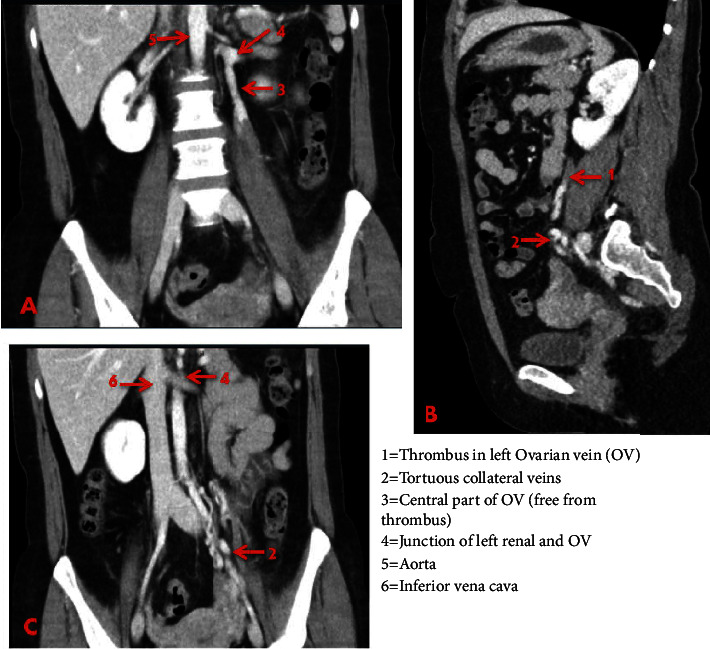
CT abdomen with contrast in venous phase showing thrombosis of the left ovarian vein and tortuous collateral veins. (A, C) coronal and (B) sagittal sections.

A young woman in her 30 s presented in the emergency room with abdominal pain in the left LQ for two weeks. The pain was shooting in character, with increasing intensity (7 of 10 on numeric pain scale) and radiation in the left flank. Clinical examination revealed mild tenderness and a palpable cord in the left LQ. A urine analysis showed isolated mild microscopic hematuria without any urological or gynecological disorders. A urine-HCG pregnancy test was negative. Blood examination was unremarkable apart from an increase of c-reactive protein to 1.69 mg/dl. After an inconclusive ultrasound and X-ray of the abdomen, a contrast (i.v.) abdominal CT was performed and revealed thrombosis of the left OV with dilated and tortuous adnexal veins (Figure 1). The thrombus extended centrally, but the confluence with the left renal vein was entirely patent. The patient had three complication-free pregnancies in the past and was on estrogen contraceptive pills. No other risk factors for deep vein thrombosis (DVT) were reported. She was admitted for monitoring and analgesia. The patient was anticoagulated with Enoxaparin 1 mg/kg twice daily and i.v analgesia with 1 g 8-hourly metamizole was administered. Estrogen contraceptives (combination of 0.1 mg levonorgestrel and 0.02 mg ethinylestradiol) were withheld and she was advised to switch over to alternative methods of contraception, as no future pregnancies were planned. Further thrombophilia testing revealed APC resistance and heterozygote mutation of factor V mutation and factor II-20210A (Table 1). A COVID-19 PCR test was negative. The patient was discharged well the next day, and anticoagulation was switched to apixaban 10 mg twice daily for one week, followed by dose reduction to 5 mg twice daily. At six months follow-up, she was free of any symptoms, and further venous CT was regarded superfluous. The patient was advised to continue the anticoagulation for another 4 weeks. A repeat urine analysis revealed no abnormalities.

## 3. Discussion

Ovarian vein thrombosis (OVT) is a rare type of thromboembolism, frequently involving the right ovarian vein (70–80%) [1]. Various retrospective case series studies report diverse risk factors associated with OVT. The common risk factors being pregnancy or puerperium in 9–81% patients [4–10], hormone therapy with estrogen (in 6–18%) [4, 10], recent surgery (in 23–100%) [4, 5, 7–10], and malignancy (in 1–100%) [4–10]. Other risk factors are recent hospitalization, pelvic inflammatory diseases, thrombophilia, and idiopathic OVT. Thrombophilia in absence of other risk factors is a rare cause of OVT. In retrospective series of 223 patients reported by Assal et al. [3], thrombophilia could be excluded in all the patients. On the contrary, Lenz et al. [4] and Rottenstreich et al. [8] reported thrombophilia in 14% and 24% patients, respectively.

The most common presenting symptom in patients with OVT is LQ abdominal pain usually on the affected side [8]. Pain radiation to the flank, upper abdomen and or groin is not uncommon. A cord like structure in the LQ is palpable in <50% of patients [1]. A high degree of suspicion is necessitated in patients with the triad of young-middle-aged female, pain in LQ, and hematuria to diagnose OVT. Ultrasound examination of the abdomen is the first line imaging modality and offers the advantages of being safe, easily available and helps rule out other abdominal pathologies. Contrast enhanced CT or CT venography offers sensitivity up to 100% and specificity up to 99% [1]. Because of ionizing radiations and risk of contrast induced nephropathy, CECT is disadvantageous in pregnant patients and patients with renal insufficiency respectively. The typical findings in a CECT are filling defects in the ovarian vein with dilated or tortuous veins as seen in Figure 1. MR venography has the benefit of being free from ionizing radiations and safe in pregnant patients but involves higher costs and is unavailable in emergency setting. After excluding other causes of OVT, thrombophilia testing should be initiated.

The diagnosis of OVT in the above-mentioned patient was achieved by CECT and thrombophilia testing, revealed APC resistance and heterozygote mutation of factor V mutation and factor II-20210A. This patient had multiple risk factors such as smoking, estrogen pills, and thrombophilia. The authors recommend thrombophilia testing for further evaluation of patient with pain abdomen of unclear etiology and OVT.

The treatment of OVT not associated with pregnancy or sepsis is predominantly based on anticoagulation. Antibiotics play a role only in case of concomitant infection for, e.g., in puerperal sepsis [11]. Rottenstreich et al. [8] reported a significant inclination towards using antibiotics in pregnancy related OVT as compared to nonpregnancy-related OVT (60 vs 21.4% *p*=0.007). The use of anticoagulation is based on the principles of the usual management of venous thromboembolism and initiated with either low molecular weight heparin (LMWH) (therapeutic dose 1 mg/kg twice daily) or unfractionated heparin. The anticoagulation is subsequently bridged to vitamin K antagonists (VKAs), which have been the standard oral anticoagulants for the past decades [3]. The recent introduction of the direct oral anticoagulants (DOACs) and their use in deep vein thrombosis and pulmonary embolism has led to their use in OVT as well. Cook et al. [12] administered 15 mg twice daily rivaroxaban, followed by 20 mg once daily indefinitely in patients with OVT with thrombophilia (factor V mutation homozygosity) and on oral contraceptive pills. Naoum et al. [13] treated pregnancy-associated OVT with tinzaparin for a week and then rivaroxaban 20 mg for 6 months.

The duration of anticoagulation in OVT remains controversial and should be based in the etiology and individual patient-associated factors. Rottenstreich et al. [8] reported a longer treatment duration in patients with nonpregnancy OVT as compared to pregnancy-associated OVT (6 vs 3 months, *p* = 0.1). Lenz et al. reported VTE reoccurrence rates of 6% at 1-year and 14.3% at 5-yearsfollow-up in OVT patients. Labropoulus et al. reported 17.4% recurrent CTE during a median follow-up of 2.3 years [2].

Based on the above facts, the authors suggest treating thrombophilia-induced OVT with anticoagulation beginning with LMWH and continuing with oral anticoagulants for lifetime. The decision of lifelong anticoagulation should be based on the etiology of OVT and individual risk profile. The aforesaid patient was administered 1 mg/kg enoxaparin daily for 1 week and then switched to direct oral anticoagulant apixaban, which was prescribed for lifetime. The patients should be reevaluated regularly for, e.g., after 3 months, 6 months, and 1 year. Another therapy option of catheter directed thrombolysis carries high risk of bleeding and is rarely required and reserved for patients with heavy thrombus progression under anticoagulation therapy or if both kidney veins are involved.

## 4. Conclusion

Even though a rare entity, a high degree of suspicion is necessitated in patients with the triad of young middle-aged female, pain in LQ, and hematuria to diagnose OVT. After ruling out the predisposing factors, testing for thrombophilia is recommended [1, 2]. Anticoagulation should be commenced with low molecular weight heparin. Switching to DOAKs is possible. Evidence on duration of anticoagulation is lacking [1] and should be based on patients' condition, symptoms, and risk factors.

## Figures and Tables

**Table 1:** Results of thrombophilia testing in the reported patient.

Analyses	Units	Reference values	Results
PTT	sec	20–40	25
Fibrinogen	mg/dl	180-350	345
Antithrombin III	%	83–118	97
Factor VIII	%	70–150	44
Protein C active	%	70– >120	68
Protein S active	%	59–118	69
Plasminogen activator inhibitor (PAI-1)	%	75–150	>120
APC resistance	NR	0.86–1.10	0.43
LA1	Sec	<44	42.2
B2-glykoprotein antibody	RE/ml	<20	5.1
Cardiolipin antibody	RE/ml	<12	<2

## Data Availability

The data used to support the findings of the study are available from the corresponding author upon request.

## Additional Points

Background: ovarian vein thrombosis (OVT) is a rare thromboembolic condition with unknown incidence in the general population. Setting: we present a case of young woman in her 30 s with left OVT. Significance: a high degree of suspicion is necessitated in patients with the triad of young middle-aged female, pain in LQ, and hematuria to diagnose OVT. Summary: OVT should be treated with anticoagulation. The duration depends on patients' risk factors.

## References

[1] Riva N., Calleja-Agius J. (2020). Ovarian vein thrombosis: a narrative review. *Hämostaseologie*.

[2] Labropoulos N., Malgor R. D., Comito M., Gasparis A. P., Pappas P. J., Tassiopoulos A. K. (2015). The natural history and treatment outcomes of symptomatic ovarian vein thrombosis. *Journal of Vascular Surgery: Venous and Lymphatic Disorders*.

[3] Assal A., Kaner J. D., Danda N., Cohen H. W., Billett H. H. (2017). Risk factors and prognosis of ovarian vein thrombosis. *Blood Coagulation and Fibrinolysis*.

[4] Lenz C. J., Wysokinski W. E., Henkin S. (2017). Ovarian vein thrombosis: incidence of recurrent venous thromboembolism and survival. *Obstetrics and Gynecology*.

[5] Gakhal M. S., Levy H. M., Spina M., Wrigley C. (2013). Ovarian vein thrombosis: analysis of patient age, etiology, and side of involvement. *Delaware Medical Journal*.

[6] Sinha D., Yasmin H., Samra J. S. (2005). Postpartum inferior vena cava and ovarian vein thrombosis—a case report and literature review. *Journal of Obstetrics and Gynaecology*.

[7] Mantha S., Sarasohn D., Ma W. (2015). Ovarian vein thrombosis after debulking surgery for ovarian cancer: epidemiology and clinical significance. *American Journal of Obstetrics and Gynecology*.

[8] Rottenstreich A., Da’as N., Kleinstern G., Spectre G., Amsalem H., Kalish Y. (2016). Pregnancy and non-pregnancy related ovarian vein thrombosis: clinical course and outcome. *Thrombosis Research*.

[9] Bhutta H. Y., Walsh S. R., Tang T. Y., Walsh C. A., Clarke J. M. (2009). Ovarian vein syndrome: a review. *International Journal of Surgery*.

[10] Covut F., Kewan T., Perez O. (2021). Direct oral anticoagulants versus warfarin and enoxaparin in ovarian vein thrombosis. *American Journal of Therapeutics*.

[11] Bannow B. T. S., Skeith L. (2017). Diagnosis and management of postpartum ovarian vein thrombosis. *Hematology*.

[12] Cook R. M., Rondina M. T., Horton D. J. (2014). Rivaroxaban for the long-term treatment of spontaneous ovarian vein thrombosis caused by factor V Leiden homozygosity. *The Annals of Pharmacotherapy*.

[13] Naoum J., Mohsen A., Daher J., Eid T. (2018). Novel management of ovarian vein thrombosis: a case report. *Saudi Pharmaceutical Journal*.

